# Laparoscopic Co-surgeon Ventriculoperitoneal Shunt Placement Versus Single Surgeon Mini Laparotomy

**DOI:** 10.7759/cureus.26057

**Published:** 2022-06-18

**Authors:** Graham Mulvaney, Michael Arnold, Caroline Reinke, Scott Wait, Mark Van Poppel, Scott McLanahan, Thomas Schmelzer, Graham Cosper, Andrew Schulman, Sarah Jernigan

**Affiliations:** 1 Neurological Surgery, Carolinas Medical Center, Atrium Health, Charlotte, USA; 2 General Surgery, Carolinas Medical Center, Atrium Health, Charlotte, USA; 3 Pediatric Surgery, Carolinas Medical Center, Atrium Health, Charlotte, USA

**Keywords:** shunt placement, hydrocephalus, mini-laparotomy, laparoscopic, co-surgeon, ventriculoperitoneal shunt

## Abstract

Introduction: Ventriculoperitoneal (VP) shunt placement is one of the most common treatments for pediatric hydrocephalus. However, device failures often occur, requiring operative revision of either the intraventricular or intraperitoneal shunt catheters. Historically, shunt placement was performed via laparotomy, but there has been a trend towards laparoscopic-assisted placement of the intraperitoneal portion of the shunt. We examined the outcomes of laparoscopic-assisted versus open VP shunt placement utilizing a local institutional retrospective review.

Methods: Single institution 2012-2017 retrospective review of all cases was performed. Patients were divided into two groups - laparoscopic and open. Thirty-day outcomes, patient age, surgery performed, surgical control time (SCT), length of stay (LOS), and readmission were analyzed.

Results: Cohort analysis inclusion criteria included 188 patients. The cohort analysis showed both decreased laparoscopic-assisted SCT (56.4 vs 32.1 min, p<0.0001) and postop complications (16.7% vs 7.1%, p<0.07). There was no significant difference in surgical site infection or readmission rates.

Conclusion: Local analysis show advantages for laparoscopic-assisted VP shunt placement over open single surgeon techniques with decreased SCT, LOS, and unplanned interventions.

## Introduction

Hydrocephalus is a significant problem in the pediatric population, occurring at a rate of 0.2-1.2 per 1000, and its management leads to 40,000 admissions a year and $2 billion in cost [1]. Ventriculoperitoneal (VP) shunt was originally described in 1908, and despite many innovations in its medical and surgical management, VP shunt remains the mainstay of treatment for this condition [2]. VP shunt placement is one of the most common treatments for hydrocephalus in children, but unfortunately still has a failure rate of up to 40% within the first year. Reoperation is frequently required to revise the intraperitoneal portion of the shunt when malfunctioning [3]. Recent studies demonstrate distal shunt malfunctions rates of 15% and shunt infection rates of 9% [4].

Given this high rate of shunt complications, innovations in distal shunt placement have been sought to minimize morbidity following VP shunt placement. Laparoscopy for placement of the peritoneal catheter was first described in the early 1990s [5]. Known benefits of laparoscopy include decreased post-operative pain and lower rates of surgical site infection [6-8] and have essentially made laparoscopy the standard of care for many abdominal operations [9].

Recently, studies have demonstrated the feasibility of laparoscopically assisted placement of the distal portion of the VP shunt [3,10]. However, there have been no large series comparing traditional open VP shunt placement by a single surgeon with laparoscopically placed VP shunt by a second pediatric surgery team. The authors of this study hypothesize that laparoscopic placement of VP shunts provides an overall benefit compared to traditional single surgeon VP shunt placement via laparotomy.

## Materials and methods

A retrospective cohort of new VP shunt placements performed at a single surgical center over a time period from 2012 to 2017 underwent chart review. Patients were included if they were under the age of 22 years and were a first-time VP shunt placement for any cause of hydrocephalus. Patients were excluded if they had incomplete data, were older than the age of 22 years, or were subdural-peritoneal shunt insertions. Although our primary focus is on postoperative patient complications, because operative time was a variable of interest, we aimed to standardize the procedural intervention as much as possible.

Patients were classified based on the surgical approach to placement of the peritoneal portion of the catheter. The open surgical cohort received a VP shunt from a neurosurgeon performing both the ventricular access and obtaining peritoneal access for the catheter using an open or mini-laparotomy in the traditional fashion. The laparoscopic cohort involved the addition of a pediatric surgeon to obtain peritoneal access and placement of the shunt catheter utilizing laparoscopic methods. Retrospectively collected perioperative data was obtained from the electronic medical record. Demographic data included sex, age, and insurance. Perioperative data included operative time, total and postoperative length of stay (LOS). Outcome measures primarily assessed complication rates, need for readmission or reoperation within 30 days, and the 90-day infection rate. Informed consent was waived after evaluation by our local investigational review board (IRB) and IRB approval is under file #11-18-28E.

Statistical analysis was performed using STATA statistics and data analysis (College Station, TX; StataCorp LLC). Statistical significance was set at p < 0.05. Confidence intervals of 95% were also used in analysis where appropriate.

## Results

Population characteristics

A total of 230 consecutive VP shunt insertions were available for review. Thirty-seven patients were excluded for incomplete data, and an additional five patients received subdural-peritoneal shunt insertion and were excluded to maintain similar technical procedure. Therefore, 188 patients met all inclusion criteria and were analyzed. A total of 90 (48%) patients underwent laparotomy with a neurosurgeon alone, and 98 (52%) patients underwent laparoscopy in conjunction with a pediatric surgeon. Patient characteristics between both groups were statistically similar (Table 1).

**Table 1:** Patient demographics for neurosurgeon alone versus combined surgeon shunt placement

Demographics	Neurosurgeon alone (N=90)	Neurosurgeon and peds surgeon (N=98)
Gender, female	55.6%	47.9%
Age in years, mean (95% CI)	5.2 (3.7, 6.7)	4.2 (3.0, 5.4)
Age range (years)	0-21	0-19.5
Insurance Status		
Medicaid/government	64.4%	62.2%
Private payor	33.3%	36.7%
Self-pay	2.2%	1.0%

Clinical outcomes

Clinical outcomes of our cohort analysis are included in Table 2. The only statistically significant difference between the two groups was a shorter surgical case time for the laparoscopic-assisted VP shunt placement. Though not statistically significant, the laparoscopic group had shorter mean postoperative length of stay and decreased complication rate compared to patients undergoing open VP shunt. However, the laparoscopic assisted cohort had a longer average total hospital length of stay but this was also not statistically significant. Length of stay for both cohorts included data from neonatal patients with initial VP shunt placement who may have had prolonged hospitalizations from associated medical comorbidities.

**Table 2:** Clinical results for neurosurgeon alone versus combined surgeon shunt placement SCT: surgical control time; LOS: length of stay

Clinical outcomes	Neurosurgeon alone (N=90)	Neurosurgeon and peds surgeon (N=98)	p-Value
SCT in minutes, mean (95% CI)	56.4 (50.6, 62.3)	32.1 (29.4, 34.8)	<0.001
LOS in days, mean (95% CI)	24 (16, 31)	27 (21, 34)	0.2
Post-operative LOS in days, mean (95% CI)	11 (8, 15)	15 (11, 20)	0.2
Any post-operative complications	16.7%	7.1%	0.07
Reoperation/readmission < 30 days	14.4%	5.1%	0.4
Infection < 90 days	2.2%	2.0%	0.9

## Discussion

The overall benefit of laparoscopic-assisted ventriculoperitoneal shunt placement as an initial surgical approach has been a contested point of discussion for several decades. Studies have previously shown the benefits of a laparoscopic-assisted approach including decreased surgical time, reduced risk of ileus, ease of access in obese patients, and decreased risk of adhesions [2,4,11,12]. Detractors of laparoscopic-assisted ventriculoperitoneal shunt placement cite the increased risk of infection, increased operative time, and difficulty in coordinating care all presumably due to the presence of an additional surgical team [13].

The cohort portion of our study is additive to the currently available literature in that we present 188 first-time ventriculoperitoneal shunt placements, performed consecutively, with direct analysis of outcomes between single-surgeon laparotomy and co-surgeon laparoscopy assisted placement. In a search of the literature, few studies have directly compared outcomes between mini-laparotomy and laparoscopic-assisted procedures [14-21]. Additionally, the data from these studies often included patients receiving revision shunt surgeries, which is less contentious having demonstrated benefit of laparoscopic-assisted ventriculoperitoneal shunt placement due to the ability to visualize adhesions, minimize shunt kinking, and optimize placement of the catheter. In our study, the only catheter with a distal malfunction was placed via laparotomy by neurosurgeon alone. Our institutional cohort analysis clearly showed a reduction in surgeon control time with laparoscopically placed VP shunts. This is most likely the result of two teams working together. Our practice is to obtain ventricular and peritoneal access simultaneously. When the neurosurgeon tunnels the distal shunt catheter, the pediatric surgeon is prepared to place the shunt intraperitoneally and has been able to perform a brief laparoscopic visualization of the abdomen to optimize peritoneal catheter placement. Our cohort analysis is limited due to small sample size, even over a five-year period, to show a statistical significance across several surgical outcomes. However, when comparing laparoscopic to open outcomes, we noted a shorter mean postoperative length of stay, decreased overall complications, and decreased incidence of reoperation within 30 days.

Our study was not designed to address the impact of laparoscopic-assisted VP shunt placement on overall costs. However, we know that many of the outcomes in our study do influence the cost of cases. While Catapano et al. were able to demonstrate a financial benefit in total hospital costs in a subgroup analysis of NPH patients [14], Gravbrot et al. recently showed evidence that directly contradicted this finding, demonstrating a statistically significant increase in costs associated with involving two surgical teams [16].

Overall, when analyzing our institutional outcomes, we see a substantial improvement in the postoperative outcomes of patients undergoing laparoscopic assisted VP shunt placement. This has become the standard practice in our institution as we believe it provides the best care for patients in our setting. 

The limitations of our study include the small sample size in our institutional cohort and variation in length of stay due to patients ranging from neonatal infants with significant medical comorbidities to patients with primary hydrocephalus without medical comorbidities that could impact length of stay. However, we believe this analysis provides a valuable addition to the literature on the benefits of laparoscopic-assisted VP shunt placement.

## Conclusions

Shunt placement is one of the most common procedures in pediatric neurosurgery. Small technical changes can significantly impact the overall outcome and some of the simplest such as optimal skin sterile prep or hand washing procedures have been evaluated in the past. We found that patients undergoing laparoscopic assisted VP shunt placement have substantially improved surgical outcomes. This includes a trend toward reduced surgeon control time, a shorter post-operative length of stay, and less than half the rate of unplanned interventions. Collaboration between pediatric neurosurgeons and pediatric surgeons facilitates care and improves overall patient outcomes.
